# An Insight into Acute Pericoronitis and the Need for an Evidence-Based Standard of Care

**DOI:** 10.3390/dj7030088

**Published:** 2019-09-02

**Authors:** Chelsea Wehr, Gianncarlo Cruz, Simon Young, Walid D. Fakhouri

**Affiliations:** 1 Center for Craniofacial Research, Department of Diagnostic and Biomedical Sciences, School of Dentistry, University of Texas Health Science Center at Houston, Houston, TX 77054, USA; 2 Department of Oral and Maxillofacial Surgery, School of Dentistry, University of Texas Health Science Center at Houston, Houston, TX 77054, USA; 3 Department of Pediatrics, McGovern Medical School, University of Texas Health Science Center at Houston, Houston, TX 77030, USA

**Keywords:** gingivitis, antibiotics, evidence-based dentistry, third molar, emergencies

## Abstract

**Background:** Pericoronitis is inflammation of the operculum associated with a partially erupted third molar. It is a highly prevalent infection of the oral cavity and presents as a painful sensation of the soft tissue encompassing the crown of the involved tooth. Though pericoronitis is common, there is no evidence-based standard-of-care for treatment of emergency patients with acute pericoronitis. **Study Design:** In this study, anonymous clinicians were asked to participate in an online survey with questions formulated to identify professional clinical background, emergency treatment preferred for acute pericoronitis, number of associated complications, frequency of third molar extraction, and patient satisfaction. **Results and Conclusion:** A statistical analysis of the collected data regarding the variance among different treatment plans and associated complications revealed little consensus in the treatment of pericoronitis. The lack of consistency of the responses focusing on the preferred treatment for emergency patients with acute pericoronitis reinforces the need for developing a standard-of-care to train future dental professionals based on well-designed randomized controlled clinical trials and meta-analyses. **Practical Implications:** The ultimate goal is developing a treatment option with the fewest complications to provide the best health care for patients with pericoronitis. This issue is seen not only as an acute infection but also has the potential to impact overall health.

## 1. Introduction

Pericoronitis is defined as an inflammation of the overlying gingiva associated with infection in the soft tissues surrounding a partially erupted tooth. Mandibular third molars are most commonly affected. This pathological condition is most prevalent in young adults, although patients of any age group may present with pericoronal inflammation. Vulnerability to this condition is substantial in the period between 16 and 30 years of age, with a maximum incidence in 21–25 years old during the most common period for a third molar eruption [1]. 

Pericoronitis is triggered by an accumulation of food debris beneath the operculum that overlaps and surrounds the partially erupted tooth, which propagates an ecological niche for a tremendous variety of polymicrobial flora, mainly consisting of anaerobic pyogenic bacteria [1,2]. Apart from the plethora of obligate facultative anaerobic microflora such as the *Streptococcus milleri* group—*Stomatococcus mucilaginous* and *Rothia dentocariosa*—anaerobic bacteria such as the *Actinomyces* and *Prevotella* species may also be present in such infection [3]. The severity of pericoronitis, whether chronic or acute, depends on a series of factors that range from the interaction of periodontal pathogens, genetic makeup that controls immune system responses, mechanical properties regarding mastication, degree of third molar impaction, and the interaction between these variable factors [1,2,3,4]. The description of pericoronitis as an “impacted third molar” is a common misconception and could prevent the development of an accurate prognosis and diagnosis, leading in some cases to an improper course of treatment plan like third molar extraction [1,4].

Despite the ubiquity of such infections in the dental field and a variety of means to effectively alleviate the pain experienced by patients, no high-level peer-reviewed clinical study has focused on the need for developing a standard-of-care for treating acute pericoronitis. This may hinder effective treatment, leading to complications and unnecessary extended discomfort [5]. Complications that most healthcare providers fear regarding this infection associated with impacted third molars are predominantly focused on spread of the infection toward fascial spaces of the neck and thoracic region, which could lead to trismus, airway obstruction, mandibular nerve injuries, and life-threatening diseases like Ludwig’s angina and sepsis [2,4,5,6].

The problem of pericoronitis goes far beyond pain of the oral cavity and may affect the individual’s productivity and quality of life. In addition to the relationship of the oral microbiome to the propagation of a severe infection such as pericoronitis, socioeconomic status also underlies various health issues regarding the oral cavity [2]. Unfortunately, the population group that suffers the most detrimental oral health infections are those that have the highest poverty rates and lowest education, resulting in limited access-to-care [7]. The fact that ten million third molars are extracted from a population of five million people in the United States with an annual cost totaling over three billion dollars illustrates the high cost of treatment for the average American [1]. Remarkably, the incidence of post-extraction mandibular fracture following third molar removal was shown to be highly associated with a history of pericoronitis and local pathological bone alterations in males over thirty-five years-old [8]. Though such post-extraction complications are very rare (less than 0.005%) [8], this illustrates the potentially serious consequences of pericoronitis and the need to find the most affordable and effective treatments for this disease. 

The null hypothesis of this study is that there is no preferred treatment modality for acute pericoronitis. Therefore, the goal of the study is to survey the preferred treatment plans for patients with acute pericoronitis, a very common disease of the oral cavity representing about 6%–9% of emergency patients annually [9], by using statistical data on treatment options obtained from our questionnaire survey. 

## 2. Materials and Methods 

### 2.1. Study Subjects and Questionnaire

An IRB approval (HSC-DB-16-0977) was obtained from the University of Texas Health Science Center at Houston guidelines (under the UTHealth System Reciprocity Agreement) on 13 December, 2016 to conduct this survey study. We contacted clinicians at 5 U.S. dental schools who agreed to participate in this study and who evaluate and treat emergency patients with acute pericoronitis as the only inclusion criteria. The questionnaire was sent to the deans or associate deans for research at each of the dental schools and was circulated by sending e-mails that had a link to an electronic anonymous survey. An invitation letter that explained the purpose and expected outcomes of this survey upfront before taking the survey was included in the questionnaire form. Around 10%–15% of the total number of clinicians at each school filled out the questionnaire of this study.

### 2.2. Medical and Dental History of Sample Collection

No patient clinical history was collected from the participants in the study. Clinicians participated in this study regardless of their ethnicity, gender, background and location. The questionnaires did not require responders to provide any identifiable data of the treated patients with acute pericoronitis.

### 2.3. Data Handling and Storage

Partial and completed responses to the anonymous survey were collected and kept encrypted on a UTHealth School of Dentistry server where only the principal investigators on this project were granted access to the data for research purposes only. All research personnel working on this study had received training on research involving human subjects, including aspects of ethical conduct, conflict of interest, privacy and confidentiality. The data will be kept for 5 years after the study is published for a follow-up purpose, and then it will be deleted from the school server. 

### 2.4. Quality Control and Assurance

All responses to our survey were considered even if the questionnaire was not fully answered. However, only complete surveys were considered in the statistical analysis in order to avoid unintentional bias. The principal investigator sent the survey on behalf of the investigators to the contact persons at the different US dental schools involved in this survey. The affiliation and identity of the clinicians participating in our survey were kept anonymous. The Qualtrics software used in this survey is secure and requires a username and password to sign in.

### 2.5. Risk Assessment and Potential Benefits

There was no risk associated with this project related to the participants and their identities. Participation was voluntary, and clinicians did not receive any direct benefit from this study. The prevalence of acute pericoronitis and current treatment plans for emergency patients were investigated. By exploring the best standard-of-care for patients based on high quality peer-reviewed publications, our aim was to investigate the preferred treatment modality for acute pericoronitis and patient satisfaction with the received treatment for this disease. All research records were kept in encrypted files. 

### 2.6. Statistical Analysis of the Collected Data 

A statistical analysis was conducted for the data collection using chi-square *p*-values. The analysis of variance among the survey categories was considered significant if a *p*-value was less than ≤ 0.05. If participants provided more than one choice for a given question, the multiple answers were included for analysis if not contradictory (i.e., “refer to a specialist” and “antibiotics immediately followed by extraction of affected tooth”).

## 3. Results

**Figure 1:**
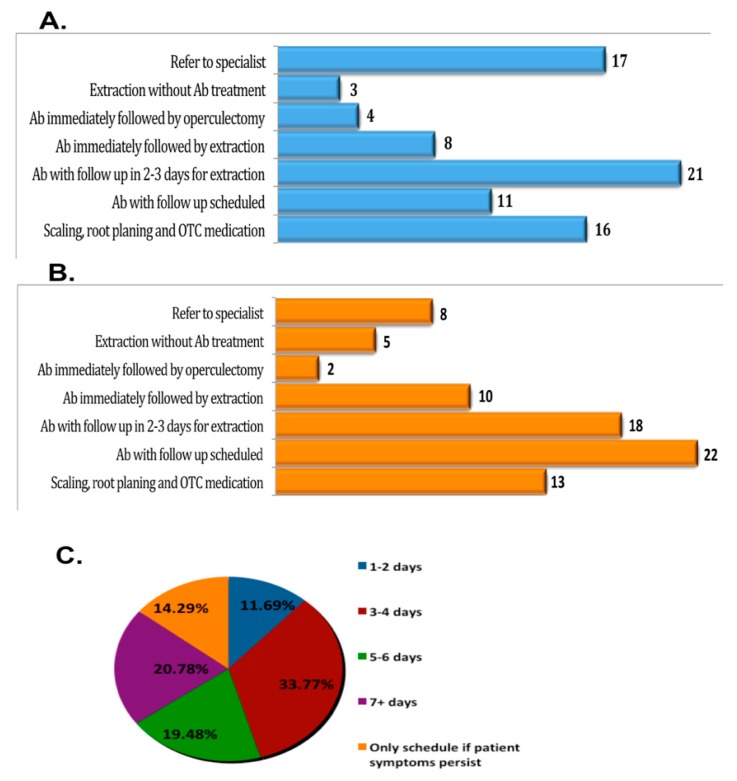
Survey results and data analysis of (**A**) providers indicate their preferred method of treatment for emergency patients presenting with acute pericoronitis of third molars. Prescribing antibiotics and scheduling a follow up appointment was reported as the most common treatment option. (**B**) Providers indicated which treatment most satisfies patient expectations on the initial emergency appointment with acute pericononitis. (**C**) The pie chart refers to the number of days to wait for a follow-up after prescribing antibiotics. The follow up timeline varied between providers. About 33.77% of dentists, a majority in this study, indicated that the preferred method of treatment for pericoronitis is antibiotic prophylaxis with follow up scheduled three-to-four days for treatment or surgery.

A total of 109 dentists participated in our study—72 (66.1%) submitted the survey complete, 23 (21.1%) submitted the survey incomplete, and 14 (12.8%) submitted the survey empty with no answers. We decided to focus our analysis on the 72 completed surveys to provide consistent statistical power to answers of each category. The data showed that the majority of the participants (43.1%) were practicing in academic settings, with most (52.8%) general dentists, and the majority of participants (66.7%) in our study having more than 15 years of experience, as shown in Table 1. On the other hand, 41.7% of the clinicians participating in our study had never performed a third molar extraction before, which raised concern about one of the common treatment options for pericoronitis (Table 1). Our results showed that there was no predominance of preferred treatment modality chosen by dentists participating in our study, where 26.4% had prescribed antibiotic therapy followed by surgery after two-to-three days. Referring to a specialist was chosen by 20.8%, and a similar percent of dentists (18.1%) choose to offer scaling and root planning as well as prescribe over-the-counter pain medications with scheduling a return visit if symptoms persisted. Impacted third molar tooth extraction without additional treatment in the first visit was not a preferred option among our dentists, as only three of the participated dentists (4.2%) selected it (Table 1). The pattern of answers by the dentists in this survey with no predominance of a preferred treatment modality exemplifies and supports our null hypothesis for a lack of standard-of-care for pericoronitis. Notably, the pattern of answers did not change even when the partially completed questionnaires were considered in the plotting of patients preferred treatment plans (Figure 1).

Regarding which treatment options had met the patient’s satisfaction, 25% of the clinicians participating in our study reported that prescribing antibiotics and scheduling a follow up visit was the treatment modality preferred by their patients. The next choice was prescribing antibiotics followed by operculectomy vs. the extraction of impacted tooth after two-to-three days in 23.6% of the patients (Table 1). Our results showed that 56% of dentists preferred prescribing antibiotics at the initial visit and scheduling a follow-up visit in two-to-three days for extraction, which was the second most common option that satisfied their patients’ expectations. About 20.8% of patients who were referred to a surgeon showed satisfaction, while 18.1% of those who preferred scaling and root planning as well as prescribing over the counter (OTC) pain medications with offering a follow up visit if no symptoms relief reported that this treatment modality fulfilled their patient expectations. The data collected in this study attested to the null hypothesis of this study and drew attention to the importance of developing a treatment protocol for acute pericoronitis that can meet the patient’s expectations while harmonizing with the preferred choice by our dentists based on high levels of scientific evidence.

It is of note that the answers provided in this study were contributed mainly by general dentists (52.8%) compared to the remaining clinicians, who are oral healthcare specialists. The skewed proportion of responses given by general dentists with a *p*-value of 0.005 may have contributed to the variable pattern of answers related to the preferred treatment protocol for patients with acute pericoronitis in this survey (Table 2). In addition, the high percentage of not performing third molar extraction by participants could be explained by the large percentage of general dentists who contributed to this study, with a *p*-value < 0.001, as described in Table 2. Hence, these data indicate the need for a better calibration of general dentists who admit patients with acute pericoronitis in their clinic but have never performed third molar extraction or operculectomy.

## 4. Discussion

Pericoronitis is a common inflammatory disease of the gingiva that overlies a partially erupted tooth, most commonly a mandibular third molar. However, the treatment of pericoronitis varies among health professionals, and no evidence-based standard of care has been established to minimize the lengthy period of oral pain and other associated complications [1,2,3,4]. This study sought to determine whether patient preference and the preferred type of treatment for patients with acute pericoronitis are impacted by the specialty of health professionals, their educational background, the clinical practice setting, and the number of years in practice. Treatment options included in the survey were as follows: Prescribing an antibiotic, the scaling and root planning of the affected area, the resection of inflamed soft tissues (operculectomy), over the counter pain medication, and/or the extraction of the impacted third molar. Notably, the wide range of techniques provided as options in the survey signifies the general lack of a standard approach or plan of action when the various specialties are confronted with this one type of infection.

Third molars (M3) are the most commonly impacted teeth, and their surgical removal is one of the most common surgical procedures performed by oral and maxillofacial surgeons and general dentists [1,10]. Impacted wisdom teeth can cause the swelling and ulceration of the gingiva around them, damage to the roots of second molars, the decay of adjacent second molars, gum and bone disease around second molars, and the development of life-threatening diseases such as Ludwig’s angina and sepsis [4,5,6]. General agreement exists that the removal of non-restorable third molars is appropriate if signs or symptoms of disease related to these teeth are present [1,4,6]. Very few reports have studied the effect of administration of nonsteroidal anti-inflammatory drugs (NSAIDs) and post-operative swelling after third molar extractions [11]. Previous studies have shown a significant reduction of trismus and facial edema when NSAIDs were used alone. The postoperative intake of ibuprofen does seem to reduce pain, facial swelling and trismus after the removal of an impacted lower third molar when compared to aceclofenac [12].

The data collected in this study enhance the underlying message of the lack of a consistent protocol-of-care for acute pericoronitis. Our results showed that 41.7% of oral health professionals had “never” performed third molar extractions, and a total of 77.8% preferred antibiotic treatment and debridement while also referring to a specialist for acute pericoronitis. Based on the responses provided by the clinicians, the survey outcomes showed that the most preferred treatment by patients was antibiotic treatment with a follow-up, and the second was antibiotic prescription with follow-up in two-to-three days for extraction after the initial visit. On the other hand, the preferred treatment by professionals was antibiotic prescription with a follow-up in two-to-three days after the initial visit, and the second was to refer to a specialist. Referral to a specialist should not be considered as a preferred treatment option, since it does not entail any treatment procedure. Furthermore, specialists might have a different opinion than simply accepting pericoronitis cases. The data collected in this study indicate there was some degree of alignment between the treatment modality preferred by the participating dentists and patient satisfaction. However, a major limitation for this conclusion is the fact that the patient responses were provided by the clinicians themselves based on their experience treating patients with acute pericoronitis. Unfortunately, other alternatives for this study were hard to accommodate in order to collect data directly from the patients themselves. That said, if patients insisted on a treatment that is considered unbeneficial or might be harmful, clinicians should follow the American Dental Association guideline, which states that “any treatment performed should be with the concurrence of the patient and the dentist. If the patient insists upon treatment not considered by the dentist to be beneficial for the patient, the dentist may decline to provide treatment. If the patient insists upon treatment considered by the dentist to be harmful to the patient, the dentist should decline to provide treatment.”

According to our study results, prescribing antibiotics and scheduling a follow-up appointment was reported to be the most common treatment option; however, the follow-up timeline varied between providers. Around 26.4% of our providers scheduled the patient for surgery two-to-three days post-initial appointment, while immediate extraction without antibiotic was not as popular among the participants in this study, with only 4.2% of respondents choosing this option. The second most common recommendation was to refer the patient to a specialist (20.8%), which suggested that dentists did not follow evidence from previous studies for odontogenic maxillofacial infections. It was concluded that extraction of the offending tooth is associated with a faster clinical and biological resolution of the infection [4]. Thus, when the causative tooth is non-restorable, it should be extracted without delay. The timing of the follow up appointment after prescribing antibiotics may be a factor in lowering the number of complications and improving the outcomes for patients experiencing pain or discomfort [2]. Thirty-three percent of our patients were scheduled for follow up after three-to-four days. The most common complication encountered in patients who had received antibiotics and were scheduled for a follow up (13.9%) was non-compliance, followed by worsening of infection.

One of the limitations of this study is the small sample size of participating health professionals (Table 1). Only 72 of the participants completed the questionnaire survey; however, they are analytically important in showing that there was no consensus for treatment of pericoronitis. Of the 72 (66.1%) of participants who completed the survey, 52.8% were general dentists, of which 43.1% practiced in an academic setting. An analysis of the collected data signifies a skewed perspective through which students may be being taught to treat this inflammatory infection that is caused by a mesio- or vertically-impacted, partially erupted third molar [13,14]. Several studies have shown a trend of a growing uncertainty for dental students surrounding the rather broad scope of oral and maxillofacial procedures, including but not limited to third molar extraction, operculectomy, and placing sutures after tooth extractions [15,16]. This addresses the issue of not obtaining adequate training due to the respondents not having sufficient preparedness or expertise to perform surgical extractions and leading to skewed reasoning for treating pericoronitis [14,15,16,17]. These studies significantly correlate with our findings regarding the statistical factor (72.3%) of having never or having only performed one third molar extraction(s) per week. This demonstrates that issues such as inflammatory disease recurrence, an extended period of oral pain, or an increase of associated cost for such infection may also be contributing factors. Hence, developing an evidence-based treatment plan for such a common health problem will help guide incoming aspiring oral health practitioners in optimizing their techniques, their calibration, and their training programs. Based on recent peer-reviewed studies with high quality evidence, it can be suggested that this could be achieved by investing in new innovative equipment and instruments or completing continuing education courses, enabling the clinician to perform curative treatment both comfortably and safely.

## 5. Conclusions and Future Perspectives

This study developed an understanding of several issues related to the lack of a standard-of-care for treating acute pericoronitis. The analysis of the survey conducted in this study indicates a need to focus on training oral health professionals and dental students on how to properly advance the proper diagnosis, prognosis, and evidence-based treatment to achieve a standard-of-care for pericoronitis. Prescribing antibiotic prophylaxis as a treatment could also lead to further complications and, potentially, a life-threatening course for the patient [18]. With a consistent show of disapproval of having third molars extracted in multiple systematic reviews [17,19], there is a need to promote collaboration amongst oral-health professionals to focus on assessing an advanced training to effectively treat this disease for general dentists and dental students. The goal is to develop a treatment option with the fewest complications through improved training for general dentists and associated oral health practitioners at US dental schools to provide the best health care for patients with pericoronitis.

## Figures and Tables

**Table 1:** Descriptive frequency of answers for selected questions in the survey.

Variables		No. (%)
**Specialty**		
	General	**38 (52.8%)**
	Pedodontics	6 (8.3%)
	Endodontics	4 (5.6%)
	Periodontics	10 (13.9%)
	Prosthodontics	2 (2.8%)
	Oral Surgery	9 (12.5%)
	Other	3 (4.2%)
**Practice setting**		
	Academic	**31 (43.1%)**
	Private	12 (16.7%)
	Hospital	1 (1.4%)
	Various settings	27 (37.5%)
	other	1 (1.4%)
**Number of years in practice**		
	<5 years	10 (13.9%)
	5–10 years	9 (12.5%)
	10–15 years	5 (6.9%)
	15+ years	**48 (66.7%)**
**How often do you perform third molar extraction**		
	Never	**30 (41.7%)**
	<1 per week	22 (30.6%)
	1–2 per week	8 (11.1%)
	3–5 per week	3 (4.2%)
	>5 per week	9 (12.5%)
**Emergency Treatment preferred for pericoronitis of third molar**		
	Scaling, root planing and OTC pain medication	13 (18.1%)
	ABT with follow up scheduled	10 (13.9%)
	ABT and extraction 2–3 days later	19 (26.4%)
	ABT and extraction at the same visit	8 (11.1%)
	ABT and Operculectomy at the same visit	4 (5.6%)
	Impacted extraction without additional treatment	3 (4.2%)
	Refer to specialist	15 (20.8%)
**Total**		**(N = 72)**

**Table 2:** Frequency of answers to survey’s question categorized based on the treatment method preferred for pericoronitis.

		Method of Treatment for Emergency Patients with Pericoronitis of the Third Molar								Total	*p*-Value
Variables		Scaling, Root Planing and OTC Pain Medication	ABT with Follow up Scheduled	ABT and Extraction 2–3 Days Later	ABT and Extraction at the Same Visit	ABTand Operculectomy at the Same Visit	Impacted Extraction without Additional Treatment	Refer to Specialist			
**Specialty**											**0.005**
	General	6	6	12	2	2	2	8		38	
	Pedodontics	1	0	1	0	1	0	3		6	
	Endodontics	1	0	0	0	0	0	3		4	
	Periodontics	3	1	4	0	1	0	1		10	
	Prosthodontics	2	0	0	0	0	0	0		2	
	Oral Surgery	0	3	1	5	0	0	0		9	
	Other	0	0	1	1	0	1	0		3	
**Practice setting**											0.875
	Academic	5	3	7	2	3	2	9		31	
	Private	2	3	2	2	1	0	2		12	
	Hospital	1	0	0	0	0	0	0		1	
	Various settings	5	4	9	4	0	1	4		27	
	other	0	0	1	0	0	0	0		1	
**Number of years in practice**											0.63
	<5 years	2	0	2	3	3	0	0		10	
	5–10 years	0	1	4	1	1	0	2		9	
	10–15 years	1	1	1	0	0	0	2		5	
	15+ years	10	8	12	4	0	3	11		48	
**How often do you perform third molar extraction**											**<0.001**
	Never	8	2	4	0	3	1	12		30	
	<1 per week	3	3	10	1	0	2	3		22	
	1–2 per week	2	1	2	2	1	0	0		8	
	3–5 per week	0	3	0	0	0	0	0		3	
	>5 per week	0	1	3	5	0	0	0		9	
